# Comparative study of ultrasonic-guided betamethasone local injection and extracorporeal shock wave therapy in post-stroke hemiplegic shoulder pain: a randomized clinical trial

**DOI:** 10.3389/fneur.2023.1158500

**Published:** 2023-07-19

**Authors:** Jingjing Zhang, Huiwen Mao, Fang Gao, Yan Li, Yang Yang

**Affiliations:** Department of Rehabilitation Medicine, Tongren Hospital, Shanghai Jiao Tong University School of Medicine, Shanghai, China

**Keywords:** ultrasonic guidance local injection, betamethasone, extracorporeal shock wave therapy, hemiplegia shoulder pain, stroke

## Abstract

**Objective:**

This study aimed to compare the efficacy and safety of ultrasound-guided local injection (UGLI) of betamethasone around the shoulder and extracorporeal shock wave therapy (ESWT) in patients with hemiplegic shoulder pain.

**Method:**

Forty-two patients with hemiplegic shoulder pain were randomly divided into the UGLI group (*N* = 21) and the ESWT group (*N* = 21). In the UGLI group, betamethasone was injected at the pain point around the shoulder under ultrasonic localization. In the ESWT group, an extracorporeal shock wave was performed at the pain points around the shoulder for 20 min of time, once a week, for 4 consecutive weeks. Both groups received rehabilitation training. The visual analog scale (VAS) evaluation was performed at baseline, 1 h, 1 week, and 1 month after treatment. Furthermore, Neer shoulder joint function scores, upper limb Fugl–Meyer assessment (FMA), modified Barthel index (MBI), Hamilton Depression Scale (HAMD), the MOS-item short-form health survey (SF-36) scores, and serum expression level of cytokine were evaluated at baseline and 1 month after treatment.

**Results:**

After 1-h treatment, the UGLI group showed a greater effect on the degree of pain than the ESWT group (*P* = 0.017). After 4 consecutive weeks of intervention, the UGLI group showed a significant improvement in the serum level of cytokine expression compared with the ESWT group (*P* < 0.05). The range of motion (ROM) of the hemiplegic shoulder (*P* < 0.05) has no difference between the two groups (*P* > 0.05).

**Conclusion:**

The ultrasonic-guided betamethasone local injection and extracorporeal shock wave both can improve hemiplegic shoulder pain. However, the UGLI can induce a more cytokine expression level.

## Introduction

Stroke is a common and refractory disease that seriously endangers human health and life safety (1). With the improvement of clinical diagnosis and treatment of stroke, the fatality rate has gradually decreased, but the survivors are always accompanied by varying degrees of dysfunction (2). Hemiplegic shoulder pain (HSP) is one of the common complications after stroke (3), which occurs within 8 weeks to 2 months after stroke, with an incidence of 16–84% (4–6). It has a great influence on rehabilitation training, function recovery, and the quality of life of patients. Therefore, to explore a more effective, safer, and convenient treatment strategy is the common goal of clinical doctors.

The mechanism of HSP is still unclear. Current rehabilitation measures for HSP include repeated transcranial magnetic stimulation (rTMS), acupuncture, neuromuscular electrical stimulation, local drug injection around the shoulder, botulinum toxin therapy, and ESWT. rTMS relieves hemiplegic shoulder pain by restoring the inhibitory–excitatory balance between the cerebral hemispheres (7), but patients with epilepsy cannot receive rTMS. Many patients cannot tolerate the strong needle feeling and refuse acupuncture treatment (8); neuromuscular electrical stimulation works slowly (9); and botulinum toxin therapy is relatively expensive (10). Previous conclusions about the clinical efficacy of local injection of steroids around the shoulder (11) and ESWT (12) on hemiplegic shoulder pain were inconsistent, and most of the previous studies were injected without accurate positioning.

Musculoskeletal ultrasound (MU) has a high resolution, which can effectively display the structures of muscles, tendons, ligaments, bursa, bones, and peripheral nerves and determine their damage (13, 14). Therefore, based on MU examination and localization, this study compared the effects of hormone injection and ESWT to improve HSP and attempted to clarify their therapeutic mechanism. We aimed to provide a scientific basis for clinical technology promotion.

## Materials and methods

### Patients

This study was approved by the ethics committee of Shanghai Tongren Hospital, Shanghai Jiao Tong University School of Medicine. This study has passed clinical trial registration (registration number: ChiCTR1800019047), including 50 patients from January 2020 to November 2020 from our Rehabilitation ward. Of the 50, eight of them were excluded (five who did not meet the inclusion criteria and three who declined to participate). The sample size was calculated using PASS 19.0 software. According to the VAS score data of the experimental group and the control group in a previous study (7), 21 cases in each group were obtained by using the two-sample independent *t*-test calculation formula. The shedding rate was 20%, and the final number of participants was 50. These patients were enrolled in this study and randomly divided into the UGLI group and the ESWT group, with 21 patients in each group by a computer-generated randomization list. All assessments in both groups were performed by a therapist who did not treat these enrolled patients and was blinded to the treatment allocation. All participants had signed informed consent before enrollment.

### Inclusion criteria

#### The inclusion criteria are as follows

(1) All patients who were diagnosed according to the World Health Organization's definition of a stroke and were confirmed by CT or MRI;
(2) First onset stroke;
(3) The patient presented with shoulder pain on the hemiplegic side within 0–6 months of onset; and
(4) MRI examination of the shoulder joint confirming the appearance of soft tissue injury of the shoulder joint.

### Exclusion criteria

#### The exclusion criteria are as follows

(1) Periarthritis of the shoulder, rheumatoid arthritis, and other diseases;
(2) Aphasia, poor cognitive function (MMSE < 23), or unable to cooperate with the treatment;
(3) Patients with basic diseases such as severe diabetes, hypertension, coronary heart disease, and poor drug control;
(4) Allergic history of steroids;
(5) Persons with blood coagulation disorder; and
(6) Those who have received local injection treatment of shoulder joints.

### Experimental procedures

The two groups received routine rehabilitation training and positioning under ultrasonic guidance. The following are specific methods:

#### Routine rehabilitation training

(a) Patients were instructed to place their good limbs and wear shoulder support when standing up to protect the affected shoulder.
(b) For 2 weeks, local ultrashort wave therapy, ultrasonic therapy, and other physical factors were used for 30 min five times week.
(c) Manual rehabilitation therapy includes painless passive shoulder movement, 15–20 shoulder movements (5–10 min) each time, during the activity, with massage of soft tissue around the shoulder joint. In the supine position, the therapist holds the shoulder blade with one hand and the shoulder with the other, moving the shoulder blade forward, outward, and upward. Then, the therapist holds the patient's hand, stretches his/her elbow straight, and pulls the his/her shoulder forward in the flexion direction. Next, while sitting, the therapist holds the upper limb on the affected side with one hand and puts the other behind the shoulder blade, stretching the upper arm forward and outward for 30 min 5 times/week for 2 weeks.

#### Positioning under ultrasonic guidance

The ultrasonic instrument was China Mindray Resona 7 ultrasonic diagnosis system, and the probe was a linear array probe with a frequency of 5–12 MHz.

**Figure 1:**
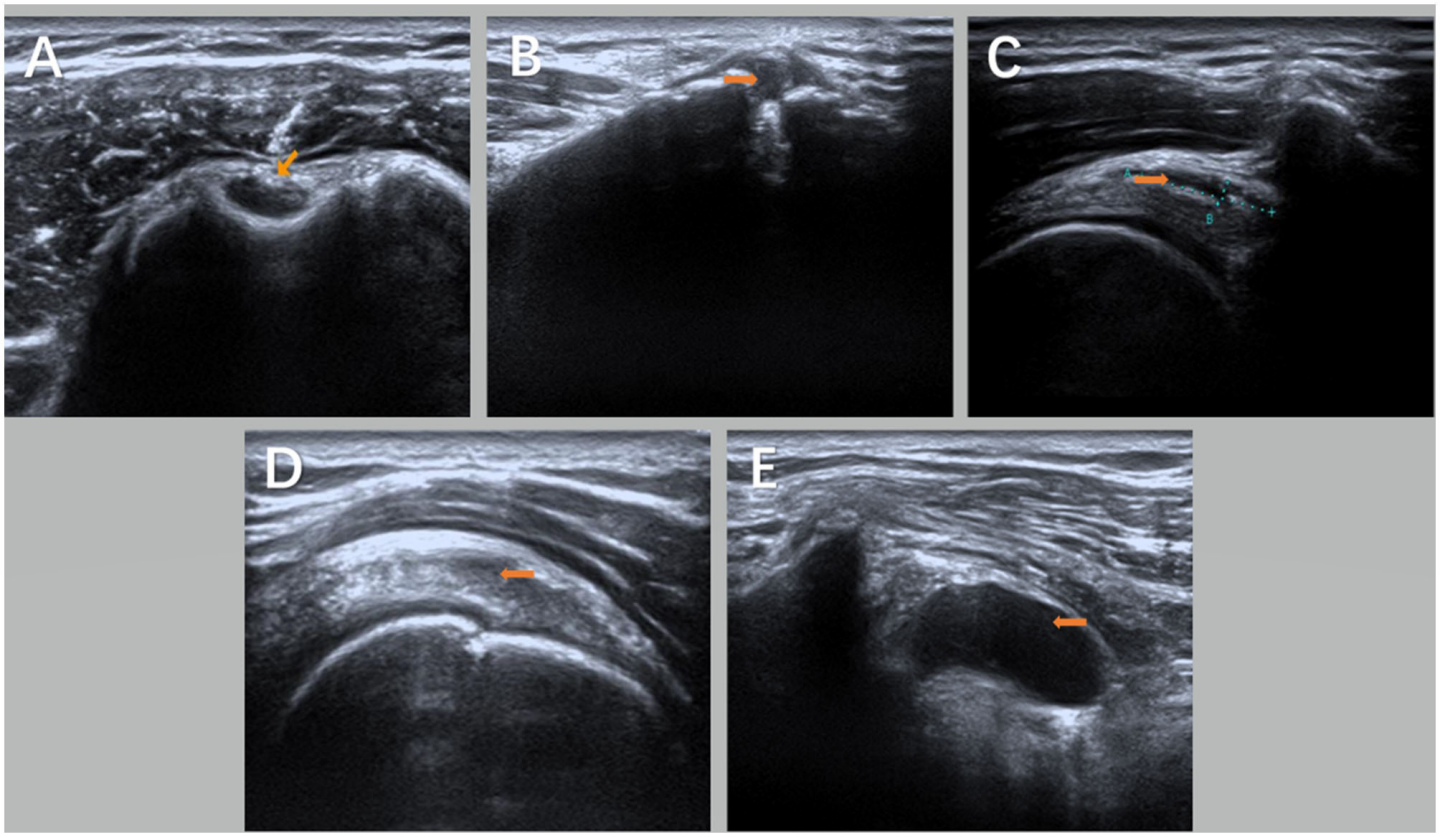
Ultrasound images of the shoulder joint on the hemiplegic side of the subject under the guidance of ultrasound [**(A)** is biceps long head tendinitis with tendon sheath effusion; **(B)** is acromioclavicular joint effusion with calcification; **(C)** is acromion-deltoid muscle Subscapular bursa effusion; **(D)** is supraspinatus tendinosis; and **(E)** is coracoid-subscapular bursa effusion]. Orange arrows show the lesion in **(A–E)**.

The patient was supine, and the shoulder injury was examined with an ultrasound. First, the long head tendon of the biceps humerus was investigated to find whether there was an injury or effusion in its tendon sheath. Second, the rotator cuff structures (supraspinatus, subscapularis, subspinatus, and teres minor) were investigated for tears and calcification, and the bursa (subacromial deltoid, coracoid process, and subscapularis bursa) were detected for fluid accumulation. Finally, the seated position was used to investigate whether there was effusion in the shoulder joint cavity and the injury of the pelvis and lip. Afterward, the damage was located and marked. The results of ultrasound imaging and magnetic resonance imaging (MRI) were consistent between the two groups (Table 1; Figure 1).

**Table 1:** Baseline characteristics of two groups.

		**UGLI (*n* = 21)**	**ESWT (*n* = 21)**	***P*-value**
Age (year)		57.19 ± 8.83	56.66 ± 9.47	0.854^*^
Medical history (day)		95.47 ± 34.86	86.9 ± 32.09	0.412^*^
Gender	Male	8 (38.1%)	12 (57.1%)	0.354^**^
	Female	13 (61.9%)	9 (42.9%)	
Stroke type	Hemorrhage	8 (38.1%)	7 (33.3%)	0.747^**^
	Infarction	13 (61.9%)	14 (66.7%)	
Hemiplegic side	Left	11 (52.4%)	9 (42.9%)	0.537^**^
	Right	10 (47.6%)	12 (57.1%)	
Comorbidities	Hypertension	9 (42.9%)	10 (47.6%)	0.756^**^
	Diabetes	5 (23.8%)	7 (33.3%)	
VAS		6.52 ± 0.98	6.14 ± 1.15	0.256^*^
Cytokines	IL-1	10.51 ± 9.35	6.15 ± 3.62	0.053^*^
	IL-6	10.67 ± 10.21	6.74 ± 3.75	0.105^*^
	IL-8	5.7 ± 4.71	5.20 ± 3.35	0.700^*^
	TNF-α	4.02 ± 2.05	2.96 ± 0.95	0.255^*^
Neer		40.14 ± 16.48	44.33 ± 11.18	0.342^*^
Barthel		61.43 ± 16.05	59.52 ± 12.23	0.668^*^
Fugl-Meyer		34.86 ± 12.56	33.38 ± 10.34	0.754^*^
HAMD		4.24 ± 2.91	2.71 ± 0.86	0.227^*^
SF-36		85.51 ± 18.09	88.64 ± 16.57	0.562^*^

^**^Chi-squared test; ^*^t-test; The *P*-value of < 0.05 was considered to be statistically significant.

#### Ultrasound-guided injection procedures

Before injection, patients and their families should be informed of the possible risks and asked to sign informed consent.

**Figure 2:**
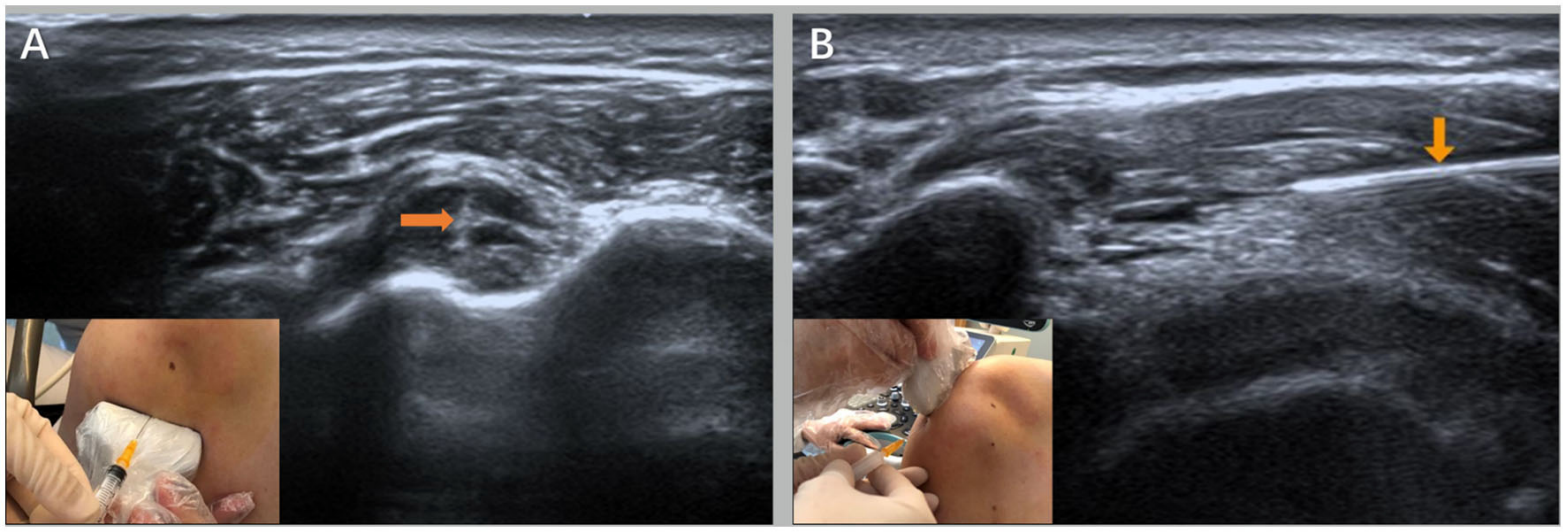
Ultrasound-guided injection operation diagram [**(A)** is the injection point of the long head of the biceps under the ultrasound guidance and the ultrasound image and **(B)** is the ultrasound-guided acromion-subdeltoid bursa injection point and the ultrasound image] as shown by the orange arrows.

Type II mucosal iodine was used to disinfect the insertion area of the long head tendon and the supraspinatus muscle of the biceps brachii three times. A disposable 5-ml syringe was used to connect the No. 7 needle to accurately pierce the tendon sheath of the long-head tendon of the biceps brachii and the area between the shoulder peak and the sliding capsule of the deltoid muscle (Figure 2). The local effusion was drained, 2.5 ml of mixed fluid—betamethasone injection (Deboisone, Schering-Plow Labo NV Belgium) 1 ml + 2% lidocaine injection (Otsuka Pharmaceutical Co., LTD., China) 1 ml + 0.9% sodium chloride injection (Hebei Tiancheng Pharmaceutical Co., LTD.) 0.5 ml—was injected after no blood was drawn back, and then, the puncture needle was pulled out. Finally, a sterile dressing covered the entry point.

To prevent infection, patients were instructed to avoid the contact of the injected site with water for 24 h after the injection. Subjects were assessed immediately after the injection, 1 week later, and 1 month later.

#### Extracorporeal shock wave therapy

Swiss DolorClast Smart was used in this study.

**Figure 3:**
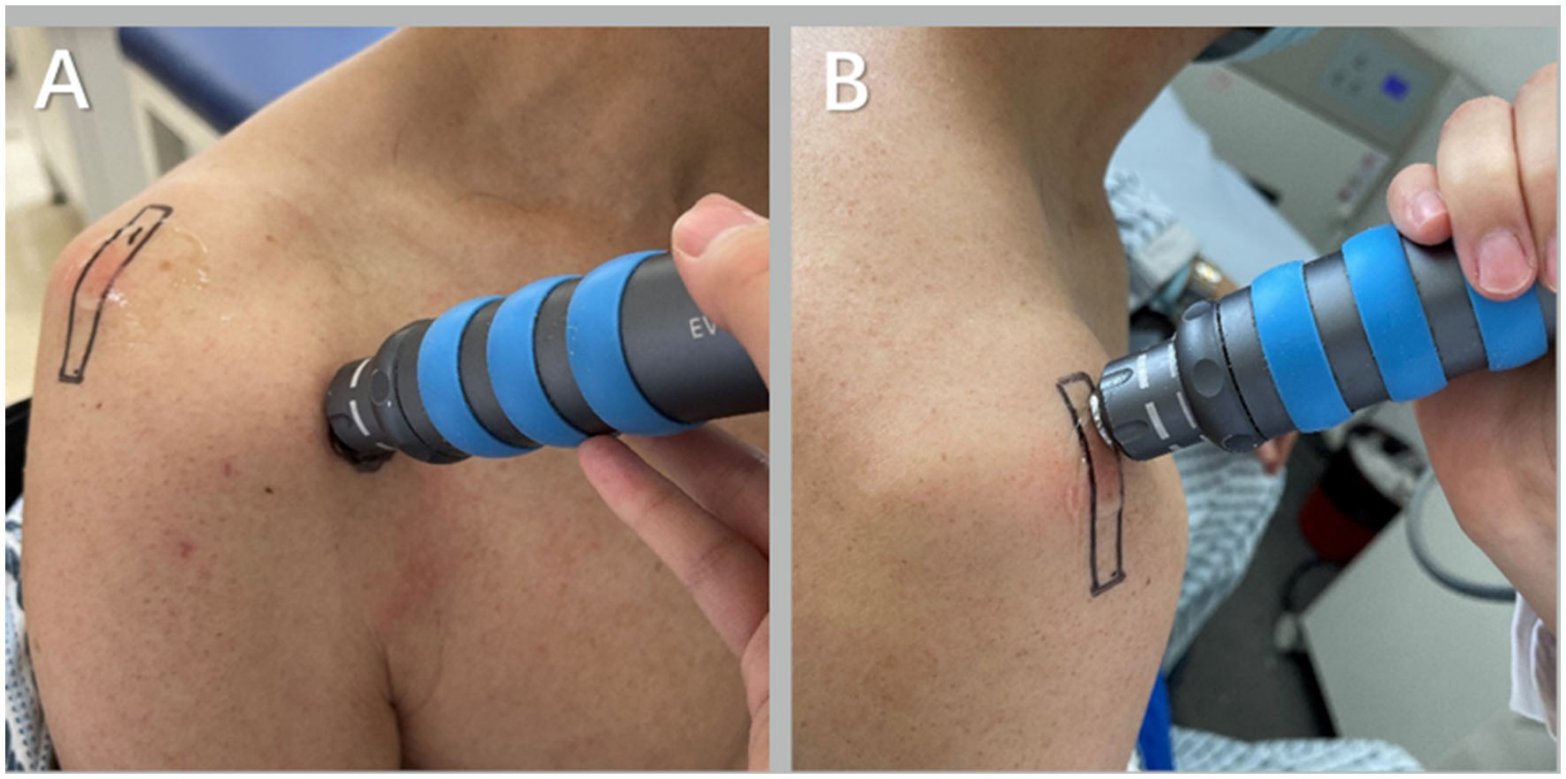
Shock wave treatment for hemiplegic shoulder pain [**(A)** is the site of the long head tendon of the biceps brachii treated by shock wave shock and **(B)** is the site of acromial-subdeltoid bursa treated by shock wave shock].

The patient was placed in the healthy lateral decubitus or a sitting position, centered on the pain point, and the coupled agent was applied at the specified position, while the shockwave treatment head was perpendicular to the surface of the tenderness point. For the patients with no obvious tenderness point or non-punctual pain, the treatment was centered on the long head of the biceps brachii (LHB) and the supraspinatus tendon (Figure 3). The impact strength was 0.12 mJ/mm2, once a week, for a total of four times. During the shockwave therapy, the therapist should inquire about the patient's symptoms, stop immediately if the pain worsens, redefine the site, monitor the skin condition after treatment, and treat any damage promptly.

### Outcome measurement

Patients were assessed at baseline and after 4-week treatment. The VAS scores were performed at baseline, 1 h, 1 week, and 1 month after intervention to assess the degree of pain. Before and 1 month after intervention, Neer score and Fugl–Meyer assessment (FMA) were used to assess the upper extremity motor function. The modified Barthel index (MBI) SF-36 assessment was adopted to assess the daily ability and the quality of life. The HAMD score assessed the emotional state, and serum inflammatory factors such as interleukin-1 (IL-1), interleukin-6 (IL-6), interleukin-8 (IL-8), and tumor necrosis factor-α (TNF-α) expression levels were detected to reflect the degree of local inflammatory reaction around the shoulder.

### Statistical analysis

All data were analyzed with SPSS 22.0 software (IBM, Inc.). The paired-sample *t*-test was used if the data were normally distributed, and the Wilcoxon signed-rank test was used if the data were not normally distributed. Gender, stroke type, hemiplegic side, and complications were compared by the chi-squared test. A *P*-value of < 0.05 was considered to be statistically significant.

## Result

### General information

Both groups were well tolerated, and no adverse event occurred during the study. During the 4-week study period, no patient dropped out.

There were 8 men and 13 women with 13 cerebral infarction and 8 cerebral hemorrhage cases in the UGLI group. Their age was 57.19 ± 8.83 years, and the disease course was 95.47 ± 34.86 days, among which nine patients had basic diseases such as hypertension and five patients had diabetes. In the ESWT group, there were 12 men and 9 women with 14 cerebral infarction and 7 cerebral hemorrhage cases. Their age was 56.66 ± 9.47 years, and the disease course was 86.9 ± 32.09 days, among which 10 patients had basic diseases such as hypertension and seven patients had diabetes.

The baseline data of the two groups included age, gender, type of stroke, and clinical courses that had no significant difference (Table 1).

### Comparison between the two groups after treatment

The VAS, Neer score, FMA, MBI, SF-36, HAMD, and serum inflammatory factors (IL-1, IL-6, IL-8, and TNF-α) had no significant difference at baseline (Table 1).

For the UGLI group, the VAS scores before and after 1-h, 1-week, and 1-month treatment were 6.52 ± 0.98, 4.38 ± 0.92, 2.71 ± 0.78, and 1.23 ± 0.89 (*P* < 0.05) and for the ESWT group, those were 6.14 ± 1.15, 5.09 ± 0.94, 3.09 ± 0.94, 0.95 ± 0.80 (*P* < 0.05), respectively. The VAS scores of the two groups were improved after treatment (*P* < 0.05). After 1-h treatment, the UGLI group was 4.38 ± 0.92, and the ESWT was 5.09 ± 0.94 (*P* = 0.017), and the UGLI group was superior to the ESWT group (Table 2).

**Table 2:** Comparison of VAS scores between the two groups.

**Group**	**Case**	**Pre-treatment**	**Post-treatment 1 h**	**Post-treatment 1 w**	**Post-treatment 1 m**
UGLI	21	6.52 ± 0.98	4.38 ± 0.92	2.71 ± 0.78	1.23 ± 0.89
ESWT	21	6.14 ± 1.15	5.09 ± 0.94	3.09 ± 0.94	0.95 ± 0.80
*P*		0.256	0.017	0.163	0.282

In terms of the serum levels of inflammatory factors (IL-1, IL-6, IL-8, and TNF-α), the scores of the UGLI group after treatment were 2.38 ± 0.66, 2.43 ± 1.29, 2.13 ± 0.75, and 1.89 ± 0.75, which was decreased compared with those before treatment, which were 10.51 ± 9.35 (*P* = 0.001), 10.67 ± 10.21 (*P* = 0.001), 5.7 ± 4.71 (*P* = 0.004), and 4.02 ± 2.05 (*P* = 0.023). The scores of the ESWT group after treatment were 3.96 ± 2.83, 5.59 ± 5.05, 3.15 ± 1.49, and 2.44 ± 0.92, which were decreased compared with those before treatment, which were 6.15 ± 3.62 (*P* = 0.001), 6.74 ± 3.75 (*P* = 0.000), 5.20 ± 3.35 (*P* = 0.005), and 2.96 ± 0.95 (*P* = 0.000), respectively. After treatment, the levels of IL-1, IL-6, IL-8, and TNF-α in the UGLI group decreased more than those in the ESWT group (*P* = 0.017, *P* = 0.008, P = 0.009, *P* = 0.040) (Table 3).

**Table 3:** Cytokine expression levels in serum of two groups.

		**UGLI (*n* = 21)**	**ESWT (*n* = 21)**	***P*-value**
IL-1, mean ± SD	Pre-treatment	10.51 ± 9.35	6.15 ± 3.62	0.053
	Post-treatment	2.38 ± 0.66	3.96 ± 2.83	0.017
		0.001	0.001	
IL-6, mean ± SD	Pre-treatment	10.67 ± 10.21	6.74 ± 3.75	0.105
	Post-treatment	2.43 ± 1.29	5.59 ± 5.05	0.008
		0.001	0.000	
IL-8, mean ± SD	Pre-treatment	5.7 ± 4.71	5.20 ± 3.35	0.700
	Post-treatment	2.13 ± 0.75	3.15 ± 1.49	0.009
		0.004	0.005	
TNF-α, mean ± SD	Pre-treatment	4.02 ± 2.05	2.96 ± 0.95	0.255
	Post-treatment	1.89 ± 0.75	2.44 ± 0.92	0.040
		0.023	0.000	

For the other scales, before and after treatment, the Neer scores of the UGLI group and the ESWT group were 40.14 ± 16.48, 73.43 ± 13.65 (*P* = 0.000) and 44.33 ± 11.18, 70.57 ± 9.79 (*P* = 0.000). The MBI values of both groups were 61.43 ± 16.05, 82.86 ± 14.28 (*P* = 0.000) and 59.52 ± 12.23, 81.19 ± 9.73 (*P* = 0.000). The FMA values of both groups were 34.86 ± 12.56, 52.33 ± 7.66 (*P* = 0.000) and 33.38 ± 10.34, 51.43 ± 5.87 (*P* = 0.000). The HAMD values of both groups were 4.24 ± 2.91, 1.29 ± 0.32 (*P* = 0.000) and 2.71 ± 0.86, 0.48 ± 0.05 (*P* = 0.000). The SF-36 values of both groups were 85.51 ± 18.09, 112.44 ± 19.12 (*P* = 0.000) and 88.64 ± 16.57, 114.35 ± 28.46 (*P* = 0.000). However, there were no significant differences between the two groups after treatment (*P* > 0.05) (Table 4).

**Table 4:** The functional improvement of the two groups of subjects.

		**UGLI (*n* = 21)**	**ESWT (*n* = 21)**	***P*-value**
Neer, mean ± SD	Pre-treatment	40.14 ± 16.48	44.33 ± 11.18	0.342
	Post-treatment	73.43 ± 13.65	70.57 ± 9.79	0.44
		0.000	0.000	
Barthel, mean ± SD	Pre-treatment	61.43 ± 16.05	59.52 ± 12.23	0.668
	Post-treatment	82.86 ± 14.28	81.19 ± 9.73	0.66
		0.000	0.000	
Fugl-meyer, mean ± SD	Pre-treatment	34.86 ± 12.56	33.38 ± 10.34	0.754
	Post-treatment	52.33 ± 7.66	51.43 ± 5.87	0.67
		0.000	0.000	
HAMD, mean ± SD	Pre-treatment	4.24 ± 2.91	2.71 ± 0.86	0.227
	Post-treatment	1.29 ± 0.32	0.48 ± 0.05	0.14
		0.000	0.000	
SF-36, mean ± SD	Pre-treatment	85.51 ± 18.09	88.64 ± 16.57	0.562
	Post-treatment	112.44 ± 19.12	114.35 ± 28.46	0.80
		0.000	0.000	

## Discussion

HSP is a common complication after stroke, and is mainly characterized by shoulder pain and limb dysfunction (15). The pathophysiological mechanism of HSP is not clear (16). Therefore, the prevention of shoulder pain and the timely and effective improvement of patients with pain symptoms will play a positive role in hemiplegia upper limb recovery. Our study aimed to compare the efficacy of UGLI and ESWT for HSP. The results showed that UGLI had a better short-term analgesic effect than ESWT. In addition, UGLI significantly reduced the serum cytokine level, thereby alleviating inflammation.

Musculoskeletal ultrasound has been widely used in the diagnosis of shoulder lesions (17–19). The sensitivity and specificity of ultrasound in the diagnosis of shoulder joint diseases are as high as 75–100 and 76–94%, respectively (20). Qixiang et al. (21) confirmed the correlation between ultrasound abnormalities and the degree of shoulder pain, and musculoskeletal ultrasound can be used as a new method for the quantitative evaluation of soft tissue lesions around the shoulder. Ultrasound-mediated treatment is simple, effective, and highly operable. According to the study of Yi et al. (22), the effective rate of “blind beating” according to experience is only 33–46%, while the effective rate of ultrasound-guided injection is 93% (20). Therefore, the musculoskeletal ultrasound was considered as a “weapon” to carry out accurate evaluation and positioning.

Previous studies have shown that corticosteroids injected into the acromial sac of HSP patients can significantly relieve shoulder pain and improve function, and our study preliminarily verifies this conclusion. Recent studies have found that the occurrence and development of shoulder joint pain are closely related to acromial slide bursitis. Moreover, inflammatory factors such as IL-1, IL-6, and TNF-α are related to pain (23) and the severity of injury (24, 25). Yamazaki et al. (26) found that inflammatory factors could be detected in the rotator cuff, acromial retrograde capsule, and serum of patients with rotator cuff injury. In our study, the local injection of hormones around the shoulder effectively reduced the levels of IL-1, IL-6, and TNF-α in the serum of HSP patients. Therefore, we suspected that the local injection of hormones around the shoulder might inhibit the local inflammatory response and reduce inflammatory symptoms by inhibiting the release of inflammatory factors.

The efficacy of ESWT for HSP mainly depends on its biological effect, namely mechanical stress and cavitation effect (27), which was also affected by shock frequency and treatment cycle. In addition, many studies (28, 29) have shown that ESWT can promote the release of substance P and prostate E2, selectively destroy some neurons, reduce the related immune response, and achieve the purpose of pain relief. The release of nitrogen oxides in the body and the formation of blood vessels promote tissue regeneration, which reduces pain by inhibiting the release of free radicals in the body. This study showed that ESWT could significantly improve shoulder pain and function in HSP patients as well as a local injection in a short time, which was consistent with the conclusions of two meta-analyses (30, 31). However, after 1-month treatment, there was no significant difference in the levels of inflammatory factors in the serum of patients in the ESWT group. To investigate the reason, the main mechanism of ESWT analgesia may increase the pain threshold (32), inhibit the excitability of nerve endings, and reduce the release of substance P and prostagenin E2 (33).

However, our study has many deficiencies, such as lower sample size and short follow-up time, the test equipment and the technology not being perfect, the number of inflammatory factors being less, and the lack of specificity. In future, we will further enlarge the sample size, have a longer follow-up period, and increase the molecular biological indicators to supplement existing conclusions.

## Conclusion

Musculoskeletal ultrasound-guided interventional therapy and ESWT can alleviate the clinical symptoms of HSP patients to a certain extent. Combined with serum inflammatory factors, ultrasound-guided local injection of hormone therapy had a better therapeutic effect.

## Data availability statement

The original contributions presented in the study are included in the article/supplementary material, further inquiries can be directed to the corresponding authors.

## Ethics statement

This study was approved by the Ethics Committee of Shanghai Tongren Hospital, Shanghai Jiao Tong University School of Medicine. The patients/participants provided their written informed consent to participate in this study.

## Author contributions

JZ and HM wrote the manuscript. FG and YL conducted the patient management. YY prepared the figures and tables. YL revised the manuscript. All authors contributed to the article and approved the submitted version.
